# A Network Analysis of Alexithymia, Interoception, Empathy, Self‐Awareness and Psychopathological Symptoms in Young People With Autism Spectrum Disorder

**DOI:** 10.1002/aur.70265

**Published:** 2026-04-28

**Authors:** Antonio Kei‐Fung Shek, Simon S. Y. Lui, Eric C. L. Lai, Raisie W. K. Wong, Jason L. F. Chan, Lok‐Yin Choi, Kimi H. Y. Lam, Eugenia Y. C. Lok, Siu‐Man Lam, Wang Yi, Raymond C. K. Chan

**Affiliations:** ^1^ Castle Peak Hospital Hong Kong Special Administrative Region Hong Kong China; ^2^ Department of Psychiatry, School of Clinical Medicine The University of Hong Kong Hong Kong Special Administrative Region, Hong Kong China; ^3^ Neuropsychology and Applied Cognitive Neuroscience Laboratory, State Key Laboratory of Cognitive Science and Mental Health Institute of Psychology, Chinese Academy of Sciences Beijing China; ^4^ Department of Psychology The University of Chinese Academy of Sciences Beijing China

**Keywords:** alexithymia, autism, empathy, interoception, network analysis, self‐awareness

## Abstract

Autism Spectrum Disorder (ASD) is associated with altered interoception, empathy, self‐awareness, and alexithymia. However, limited research has been conducted to investigate the interrelationships of these constructs with psychopathological symptoms. A prior network analysis in college students examined the interrelationship of these constructs and demonstrated that cognitive empathy and alexithymia influenced interoception and autistic features. We aimed to examine the interrelationships of these constructs in people with clinical ASD using network analysis. We recruited 208 young people aged 15–25 with Autism Diagnostic Observation Schedule (ADOS‐2) confirmed diagnosis of ASD and administered self‐report measures for interoception, empathy, self‐awareness, and alexithymia. We constructed a regularized partial correlation network. The results showed the alexithymia node of “difficulty‐describing‐feelings‐to‐others” (expected influence (EI) = 0.854), interoceptive awareness (strength = 1.273; EI = −0.084), depressive symptoms (EI = 0.537), and anxiety symptoms (EI = 0.652) were important nodes. Our findings suggested that alexithymia, depressive, and anxiety symptoms influenced empathy, self‐awareness, and autistic features in people with ASD and supported the important role of interoception in the network.

## Lay Summary

- Autistic people have diverse symptoms, such as difficulty‐to‐articulate emotion, low awareness of inner‐bodily signals, empathic difficulty, low self‐awareness, anxiety and depression.
- These symptoms mutually interact with each other, forming a symptom network.
- We assessed this complex symptom network in 208 autistic people aged 15–25.
- Difficulty‐to‐articulate‐emotion, anxiety, and depressive symptoms are important in bringing changes to the network.
- Low awareness of inner‐bodily signals is also important, because it connects most strongly with all the remaining symptoms in the network.

## 1 Introduction

Autism Spectrum Disorder (ASD) is a neurodevelopmental disorder affecting 1 in 100 children worldwide (Zeidan et al. 2022). Although ASD reflects human neurodiversity (Hunt and Procyshyn 2024), this disorder is associated with a heterogeneous set of co‐occurring traits and symptoms, leading to functional impairments. Beyond the core diagnostic criteria described in the DSM‐5 (American Psychiatric Association 2013), several psychological and neurosensory constructs, such as emotional processing (including the abilities to understand, express, and share emotions), self‐awareness, and perception of one's own internal bodily states (interoception) are particularly implicated in the ASD population. Besides, ASD is associated with increased risk of co‐morbid anxiety and depressive disorders (Kirsch et al. 2020; Lai et al. 2019). Co‐occurring anxiety and depressive symptoms in ASD can aggravate emotion dysregulation (Gotham et al. 2015). Clarifying the co‐occurring patterns of these traits/symptoms and autistic features in people with ASD can bring useful insights to identify potential intervention targets to improve clinical outcomes.

Alexithymia refers to the inability to identify, describe, and express emotions (Bagby et al. 1994), and is an important facet of emotion processing (Preece and Gross 2023). It has been proposed that impaired awareness of one's emotion (such as alexithymia) can impede adaptive strategies and promote maladaptive strategies to regulate emotion, leading to anxiety and depressive symptoms (Cai et al. 2018). ASD individuals with high levels of alexithymia have more severe social communication deficits and anxiety symptoms (Milosavljevic et al. 2015; Oakley et al. 2020). Alexithymia is believed to mediate the relationship between autistic features, depressive, and anxiety symptoms in adults with ASD (Morie et al. 2019). Empathy refers to the ability to understand and share emotions with others (Fletcher‐Watson and Bird 2019), promoting prosocial behavior (Rieffe et al. 2021). Impaired empathy is a heritable latent trait of ASD (Baron‐Cohen and Wheelwright 2004), such as toddlers who subsequently developed ASD having showed early impairments in “empathic responding” to parental distress at 2–3 years of age (McDonald and Messinger 2011). Importantly, alexithymia and empathy are interrelated in people with ASD (Bird et al. 2010), and alexithymia can aggravate empathy deficits in the clinical ASD population, leading to social impairments (Bird and Viding 2014).

The ability to process internal bodily signals has attracted growing attention (Garfinkel et al. 2016). Interoception refers to the ability to sense, interpret, and integrate internal bodily signals, such as heartbeats, breathing, temperature, muscle tension, gastric discomfort, and intestinal tension (Craig 2002). It has been theorized that interoceptive sensitivity is the basis for developing abilities to understand and regulate one's own emotion and to infer others' emotions (Quattrocki and Friston 2014). Besides, people with ASD have difficulty in objectively detecting bodily signals but would over‐inflate subjective perception of bodily sensations. This pattern of altered interoception may increase likelihood for anxiety symptoms (Garfinkel et al. 2016). Altered self‐awareness is commonly found in people with ASD (Yoshimura and Toichi 2014) and associated with impaired interoception (Seth and Tsakiris 2018; Palmer and Tsakiris 2018).

Two theoretical frameworks concerning the co‐occurrence of these traits in ASD are noteworthy. The alexithymia hypothesis (Bird and Cook 2013) posits that social‐affective symptoms are related to co‐occurring alexithymia, rather than an integral part of the phenotype of ASD; and interoceptive impairments can be explained by alexithymia. The empirical study conducted by Shah et al. (2016) supported this notion, by demonstrating that alexithymia (not autism) was linked to impaired interoception and empathy. On the other hand, the oxytocin‐interoception hypothesis (Quattrocki and Friston 2014) posits that altered oxytocin system in ASD is the cause of the impaired interoception, which in turn leads to the alexithymia, impaired empathy and other social‐affective symptoms. This hypothesis is not consistently supported by empirical findings. For instance, people in ASD may have intact interoception (Nicholson et al. 2018; Butera et al. 2023); and people with alexithymia can exhibit either high or low interoceptive abilities (Scarpazza et al. 2022). While conflating the different dimensions of interoception (e.g., interoceptive accuracy, interoceptive sensibility, and interoceptive awareness) (Garfinkel et al. 2016) as a single construct might have resulted in the previous inconsistent findings (Mul et al. 2018), the complex pattern of co‐occurring traits such as interoception, alexithymia, empathy, and social‐affective symptoms might not have been adequately addressed using traditional statistical methods. Moreover, although alexithymia has been found to mediate the relationship between interoceptive abilities, and cognitive and affective empathy (Mul et al. 2018), few previous research have studied alexithymia, empathy, interoception and autistic features simultaneously in clinical ASD samples.

Network analysis (Borsboom and Cramer 2013; Borsboom 2017; Epskamp et al. 2017) applies regularized partial correlations to model the mutual and reciprocal influences between different variables (nodes). This data‐driven, atheoretical analysis is a useful method to study complex interrelationships. A recent network analysis (Lee et al. 2023) has examined the interplay between autistic features, irritability, and executive functions in a combined sample of children and adolescents (mean age = 14.31) with ASD, their unaffected siblings, and neurotypical peers. The results showed that these domains were weakly correlated in the network (Lee et al. 2023). Importantly, a previous network analysis (Yang et al. 2022) has examined the inter‐relationship between emotion expression, empathy, interoception, and autistic features in a 1360 College student sample, among which 408 are autistic people as defined by the Autism‐Spectrum Quotient scale (Baron‐Cohen et al. 2001). The results suggested that alexithymia had the highest centrality indices in the network (Yang et al. 2022). However, its generalizability to clinical ASD population remains unclear. Moreover, anxiety and depressive symptoms could interact with alexithymia and autistic features (Milosavljevic et al. 2015; Oakley et al. 2020; Morie et al. 2019) but were not included as nodes (Yang et al. 2022). It remains unclear whether psychopathological symptoms would interact with interception, empathy, and self‐awareness in clinical ASD population.

To address the aforementioned shortcomings, this study aimed to examine the complex inter‐relationship between alexithymia, interoception, empathy, self‐awareness, autistic features, and psychopathological symptoms in clinical ASD populations. This study also aimed to extend the prior relevant findings in nonclinical samples (Yang et al. 2022), and to identify the central node in the network, by estimating the centrality indices. In the light of previous results, we hypothesized that (1) alexithymia would have high values of centrality indices in a clinical sample with ASD (Yang et al. 2022); (2) alexithymia would be linked with interoceptive awareness (Shah et al. 2016) and empathy (Mul et al. 2018); and (3) anxiety and depressive symptoms would be linked with alexithymia (Milosavljevic et al. 2015; Morie et al. 2019; Oakley et al. 2020) as proposed by the emotion dysregulation model of ASD (Cai et al. 2018).

## 2 Method

### 2.1 Participants and Procedure

We recruited 208 young people with DSM‐5 ASD from outpatient psychiatric clinics at Castle Peak Hospital and Tuen Mun Hospital, Hong Kong, and from the Support Centers for Persons with Autism (SPAs), from August 2023 to May 2024. The SPAs are public‐funded community centers to provide training and support to young people with ASD in Hong Kong. The inclusion criteria included (1) aged 15–25, (2) ethnic Chinese, and (3) able to communicate in written and oral Chinese. The exclusion criteria included (1) personal history of DSM‐5 psychotic disorders, (2) history of substance use in the past 12 months, (3) mental retardation or IQ < 70, (4) history of neurological disorder or head injury with loss of consciousness for > 30 min, and (5) severe (uncorrected) hearing or visual impairment.

All participants received structured interviews with qualified psychiatrists for diagnostic ascertainment. Participants' medical records were retrieved to gather demographics and clinical information. Participants completed the self‐report measurements during the face‐to‐face interviews with psychiatrists. Cash coupons valued HK$50 (approximately US$6.5) were provided to each participant upon the completion of this study. The research study had been prospectively reviewed and approved by the Clinical Research Ethics Review (CRER) of the Hospital Authority Hong Kong (Protocol number: PAED‐2023‐047). Participants aged > 18 and the parent/guardian of participants aged < 18 provided written informed consent. The authors assert that all procedures contributing to this work comply with the ethical standards of the relevant national and institutional committees on human research and with the Helsinki Declaration of 1975, as revised in 2008.

### 2.2 Measurements

Trained psychiatrists administered the Autism Diagnostic Observation Schedule, second edition (ADOS‐2), Module 4 to participants. The ADOS‐2 is a diagnostic interview for ASD (Lord et al. 2012), and has been applied to the Chinese setting with high validity and reliability (So et al. 2021; Chiu et al. 2024; Chung et al. 2023). We calculated the Social Affect subscale and the Restricted & Repetitive Behaviors subscale scores of ADOS‐2 Module 4 (Hus and Lord 2014), which confirmed the diagnosis of ASD. Moreover, we administered the Chinese version of the 50‐item, self‐report Autism‐Spectrum Quotient (AQ) (Baron‐Cohen et al. 2001; Lau et al. 2013) to measure participants' social skills, attention switching, attention‐to‐detail, communication and imagination. The Chinese versions of the 21‐item, self‐report Beck Depression Inventory (BDI) (Beck et al. 1961; Shek 1990) and the 21‐item, self‐report Beck Anxiety Inventory (BAI) (Beck et al. 1988; Pang et al. 2019) were used to measure depressive and anxiety symptoms, respectively.

Participants completed four additional self‐report scales. First, the Chinese version of the 32‐item Multi‐Dimensional Assessment of Interoceptive Awareness (MAIA) was used (Mehling et al. 2012; Lin et al. 2017). Each item is rated on a 6‐point Likert scale. The MAIA contains eight subscales, namely Noticing, Not‐distracting, Not‐worrying, Attention regulation, Emotional awareness, Self‐regulation, Body listening, and Trusting. Higher MAIA total scores indicate better interoceptive awareness. Second, participants completed the Chinese version of the 20‐item Toronto Alexithymia Scale (TAS‐20) (Bagby et al. 1994; Zhu et al. 2007). Each item is rated on a 5‐point Likert scale. The TAS‐20 generates three factors, that is, the difficulty identifying feelings and distinguishing them from the bodily sensations of emotions (DIF) (seven items), difficulty describing feelings to others (DDF) (five items), and externally oriented cognitive style of thinking (EOT) (eight items) (Zhu et al. 2007). Higher scores indicate more severe alexithymia. Third, the Chinese version of the 17‐item Self‐Consciousness Scale (SCS) was used (Fenigstein et al. 1975; Shek 1994). Each item is rated on a 5‐point Likert scale. The SCS taps into private self‐consciousness, public self‐consciousness, and social anxiety. Higher SCS total scores indicate higher levels of self‐consciousness. Lastly, participants completed the Chinese version of the 31‐item Questionnaire of Cognitive and Affective Empathy (QCAE) (Reniers et al. 2011; Liang et al. 2019). Each item is rated on a 4‐point Likert scale. The QCAE generates two subscales, namely the cognitive and the affective empathy subscales. Higher subscale scores indicate better empathy.

### 2.3 Data Analysis

Statistical Package for the Social Sciences (SPSS) version 26 was used for data management and descriptive analysis. The mean and standard deviation of all variables were calculated. The gender differences in these variables were examined using independent sample *t*‐tests. The significance level was set at *p* = 0.05. The R version 4.4.1 package qgraph (Epskamp et al. 2012) was used to construct regularized partial correlation networks. Considering our sample size and the sample‐to‐variable ratio (Ávalos‐Tejeda and Calderón 2025), we included 10 variables (nodes) in the network, namely the AQ‐total score, BAI, BDI, MAIA‐total score, cognitive empathy, affective empathy, SCS, TAS‐DDF, TAS‐DIF, and TAS‐EOT. As such, we did not use the subscale scores of the MAIA and the SCS as nodes in the main analyzes. However, it is plausible that different dimensions of interoception and autistic features would have differential relationships with empathy, alexithymia and psychopathological symptoms. We therefore further conducted *supplementary* analyzes, by including all the subscale scores of our measurements in a regularized partial correlation network, at the expense of reduced statistical power. We performed Kolmogorov–Smirnov test to examine the normality distribution of the variables, and nonparanormal transformation was applied if necessary (Epskamp and Fried 2018). We estimated the regularized partial correlations (edge) between two nodes, while controlling for the effects of all other nodes. To maintain network sparsity and prevent overfitting, the Least Absolute Shrinkage and Selection Operator (LASSO) was employed, which penalized smaller partial correlations by effectively shrinking them to zero (Friedman et al. 2007). Moreover, the Extended Bayesian Information Criterion (EBIC) was used in conjunction with LASSO to select a more “parsimonious model,” which balanced simplicity and explanatory power (Chen and Chen 2008). The network was displayed according to the Fruchterman and Reingold algorithm (Fruchterman and Reingold 1991) for visualization, which placed highly interconnected nodes at the center, and lesser‐connected nodes at the periphery.

### 2.4 Network Centrality

We calculated the centrality indices of expected influence (EI), strength, closeness, and betweenness for each node (Opsahl et al. 2010; Robinaugh et al. 2016; Epskamp et al. 2017). The index of strength refers to the sum of the correlations of a given node with all other nodes, reflecting how well the node is directly connected to other nodes. The EI resembles strength, but it retains the positive or negative value of the edge weights, thus reflecting the potential impact of a given node on the entire network. The index of betweenness refers to the number of times a given node lies on the shortest path between any other two nodes, reflecting the node's significance in linking other nodes. Lastly, the index of closeness indicates how close a given node would be to all other nodes in the network. It was calculated as the inverse of the weighted sum of distances from the node to all others, and therefore can reflect how well the node is indirectly connected to other nodes.

### 2.5 Network Accuracy and Stability

Following Epskamp et al. (2017)'s guidelines, we calculated the 95% confidence intervals (CIs) of edge weights, using the “bootstrapping function” in the R package bootnet. It involved randomly sampling the dataset with replacement to create new resampled datasets, and then calculating the edge weights on each of these resampled datasets. We repeated 2500 times of bootstrapping for each edge. Besides, we calculated the correlation stability (CS) coefficients of centrality indices, using the “case‐dropping subset bootstrapping” procedures. The bootstrapping procedures involved repeated estimations of the value of the centrality indices using numerous subsets of the data, with an increasing proportion of participants removed. The CS coefficients indicate the maximum proportion of cases/participants that could be removed, while retaining a correlation of at least 0.7 between the original and subsample‐derived centrality indices, with 95% certainty. A CS coefficient of > 0.5 is considered sufficient, and a value reaching 0.75 is considered excellent (Epskamp et al. 2017).

## 3 Results

### 3.1 Descriptive Statistics

Table 1 shows the sample characteristics. All participants (*N* = 208, mean age = 18.6, SD = 2.9) were having active psychiatric follow‐up for ASD. As expected, 72.1% of the sample were male, and 18% had comorbid anxiety disorder or depression. Around 16% of participants had received psychotropic medications. Table S1 shows the comparisons between male (*n* = 150) and female (*n* = 58) participants. The two gender groups did not differ in autistic symptom profile and severity, as measured by the ADOS‐2 Social Affect subscale and the Restricted & Repetitive Behaviors subscale scores, and the AQ subscale scores (*p*s > 0.05). The two groups also showed similar ratings on interoception, alexithymia and self‐consciousness (*p*s > 0.05). We found a significant gender difference in affective empathy (*t* = −3.101, *p* = 0.002) but not cognitive empathy (*p* > 0.05), with female participants having better affective empathy than male participants. In light of comparable ratings between male and female participants, and the small sample of female participants with ASD, we did not build a gender‐stratified network, nor carry out network comparison tests.

**TABLE 1:** Sample characteristics.

	ASD participants (*N* = 208)			Kurtosis	*p* (KS test)
	Mean	SD	Skewness		
Age (years)	18.57	2.890			
Gender (male vs. female)	150 versus 58				
Recruitment site (clinics vs. SPA, *n*)	172 versus 36				
ADOS‐2 SA subscale	8.12	1.119	0.668	−0.619	< 0.001
ADOS‐2 RRB subscale	3.60	0.787	0.189	0.095	< 0.001
AQ‐social skill	6.01	2.728	−0.235	−0.845	< 0.001
AQ‐attention switching	6.62	1.798	−0.454	−0.398	< 0.001
AQ‐attention to details	5.91	2.201	−0.224	−0.276	< 0.001
AQ‐communication	5.69	2.386	−0.270	−0.621	< 0.001
AQ‐imagination	5.53	2.527	0.103	−0.616	< 0.001
TAS‐DDF	16.41	4.796	−0.209	−0.738	0.001
TAS‐DIF	21.91	7.339	−0.263	−1.066	< 0.001
TAS‐EOT	22.66	5.484	−0.42	−0.241	< 0.001
SCS score	36.50	11.374	−0.498	−0.631	< 0.001
MAIA score	76.95	31.635	−0.041	−0.744	0.018
MAIA‐noticing	2.91	1.259	−0.383	−0.687	< 0.001
MAIA‐not distracting	2.20	0.894	0.205	0.092	< 0.001
MAIA‐not worrying	1.85	1.194	0.707	−0.093	< 0.001
MAIA‐attention regulation	2.33	1.318	0.135	−0.871	0.003
MAIA‐emotion awareness	2.74	1.249	−0.163	−0.932	0.001
MAIA‐self regulation	2.18	1.327	0.046	−0.898	< 0.001
MAIA‐body listening	2.30	1.406	0.164	−0.794	0.012
MAIA‐trusting	2.52	1.472	−0.120	−1.031	< 0.001
QCAE‐cognitive	47.47	12.470	0.113	−0.541	0.007
QCAE‐affective	27.52	6.406	−0.299	−0.784	< 0.001
BDI	16.05	10.884	0.679	−0.042	< 0.001
BAI	15.99	11.596	0.517	−0.567	< 0.001

Abbreviations: ADOS‐2 Module 4 score = Autism Diagnostic Observation Schedule Second Edition calibrated severity score; ADOS‐2 SA = The Autism Diagnostic Observation Schedule Second Edition Module 4‐Social Affect subscale; ADOS‐2 RRB = The Autism Diagnostic Observation Schedule Second Edition Module 4‐the Restricted & Repetitive Behaviors subscale; AQ = Autism‐Spectrum Quotient; AQ‐social skill = The Autism‐Spectrum Quotient‐social skills scale; AQ‐attention switching = The Autism‐Spectrum Quotient‐attention switching scale; AQ‐attention to details = The Autism‐Spectrum Quotient‐attention‐to‐detail scale; AQ‐communication = The Autism‐Spectrum Quotient‐communication scale; AQ‐imagination = The Autism‐Spectrum Quotient‐imagination scale; BDI = The Beck Depression Inventory; BAI = The Beck Anxiety Inventory; KS test = Kolmogorov–Smirnov test; MAIA = The Multidimensional Assessment of Interoceptive Awareness; MAIA‐noticing = The Multidimensional Assessment of Interoceptive Awareness‐Noticing subscale; MAIA‐not distracting = The Multidimensional Assessment of Interoceptive Awareness‐Not‐distracting subscale; MAIA‐not worrying = The Multidimensional Assessment of Interoceptive Awareness‐Not‐worrying subscale; MAIA‐attention regulation = The Multidimensional Assessment of Interoceptive Awareness‐Attention regulation subscale; MAIA‐emotion awareness = The Multidimensional Assessment of Interoceptive Awareness‐Emotional awareness subscale; QCAE‐cognitive = The Questionnaire for Cognitive and Affective Empathy‐cognitive empathy subscale; QCAE‐affective = The Questionnaire for Cognitive and Affective Empathy‐affective empathy subscale; SCS = The Self‐Consciousness Scale; TAS‐DDF = The Toronto Alexithymia Scale‐Difficulty Describing Feelings subscale; TAS‐DIF = The Toronto Alexithymia Scale‐Difficulty Identifying Feelings subscale; TAS‐EOT = The Toronto Alexithymia Scale‐Externally‐Oriented Thinking.

### 3.2 Network Estimation

**FIGURE 1:**
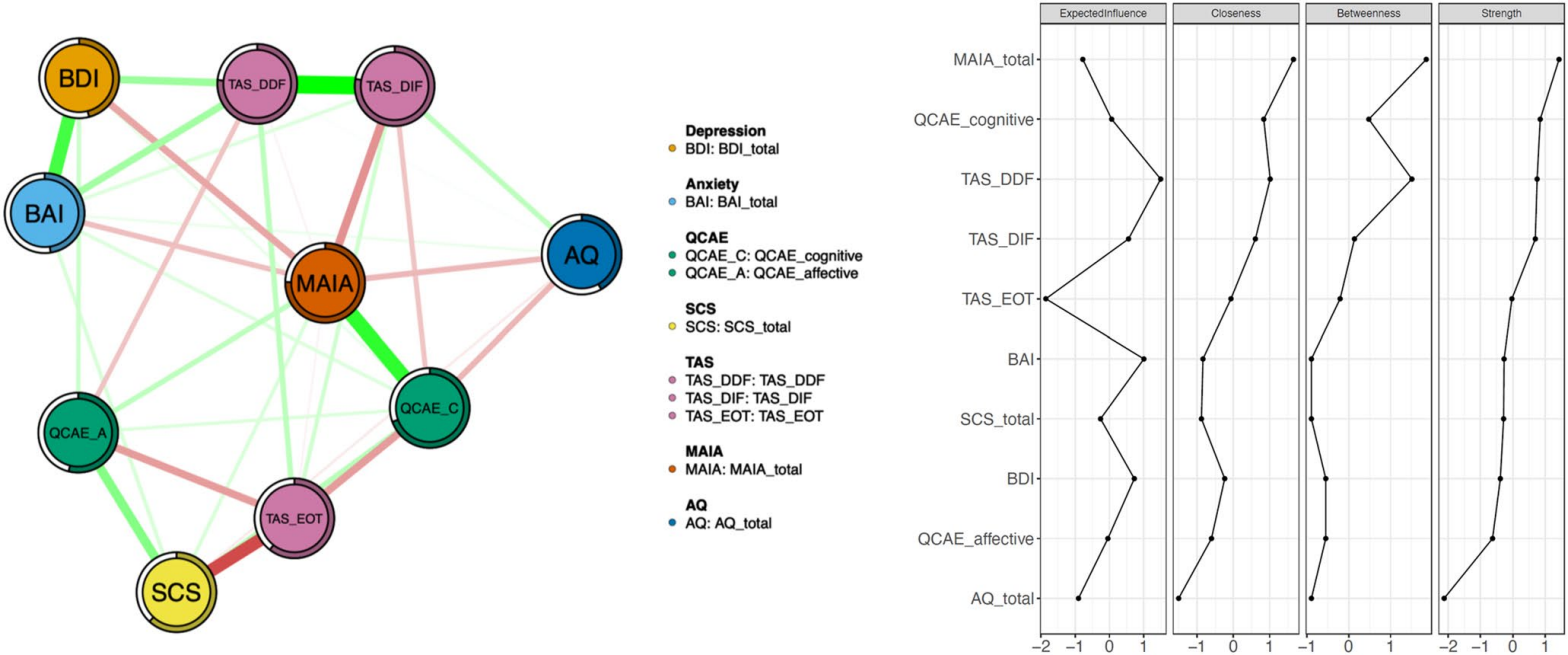
Regularized partial correlation network and centrality indices in young adults with ASD (*N* = 208). The green lines are positive associations. Red lines are negative associations. Thickness and saturation of lines are the strength of associations. The filled part of the circle around each node is the predictability. AQ = Autism‐Spectrum Quotient; BAI = The Beck Anxiety Inventory; BDI = The Beck Depression Inventory; MAIA = The Multidimensional Assessment of Interoceptive Awareness; QCAE‐affective = The Questionnaire for Cognitive and Affective Empathy‐affective empathy subscale; QCAE‐cognitive = The Questionnaire for Cognitive and Affective Empathy‐cognitive empathy subscale; SCS = The Self‐Consciousness Scale; TAS‐DIF = The Toronto Alexithymia Scale‐Difficulty Identifying Feelings subscale; TAS‐DDF = The Toronto Alexithymia Scale‐Difficulty Describing Feelings subscale; TAS‐EOT = The Toronto Alexithymia Scale‐Externally‐Oriented Thinking.

Figure 1 shows the regularized partial correlation network. On inspection, all the nodes were densely connected. The BDI and BAI nodes (regularized edge = 0.356) were strongly connected, as were the TAS‐DDF and TAS‐DIF nodes (regularized edge = 0.489). The MAIA node was strongly linked with cognitive empathy (regularized edge = 0.340); the node of SCS was negatively correlated with TAS—the node of EOT (regularized edge = −0.345). The TAS‐DIF node was linked to multiple nodes in the network, with a strong and positive link with the TAS‐DDF node (regularized edge = 0.489), a strong and negative link with the MAIA node (regularized edge = −0.209), and a negative link with cognitive empathy (regularized edge = −0.113). The BDI and the BAI nodes were positively correlated with the TAS‐DIF (regularized edge = 0.023, regularized edge = 0.066) and the TAS‐DDF (regularized edge = 0.187, regularized edge = 0.180) nodes, implicating that higher levels of alexithymia in identifying and describing feelings were associated with more depressive and anxiety symptoms in young people with ASD. Notably, the AQ node was negatively correlated with cognitive empathy (regularized edge = −0.140), the MAIA node (regularized edge = −0.136), and the SCS node (regularized edge = −0.038), implicating that autistic features were associated with more difficulty in cognitive empathy, interoception, and self‐awareness. Moreover, the AQ node was positively correlated with the TAS‐DIF (regularized edge = 0.124) and weakly connected with the TAS‐DDF (regularized edge = 0.039), implicating the association between autistic features and alexithymia. Table S2 shows the regularized partial correlation coefficients of all the edges in the network; whereas Figure S1 shows the bootstrapped 95% CIs of edges.

The regularized partial correlation network using all the subscales of the measurements as nodes is shown in Figure S2. In brief, this *supplementary* network suggested that the different dimensions of interoception, empathy, alexithymia, and autistic features were densely connected with self‐awareness, anxiety, and depressive symptoms. However, the *supplementary* results should be interpreted with caution due to the sample size limitation.

### 3.3 Centrality Indices

As shown in Figure 1, the TAS‐DDF node (EI = 0.854) had the highest EI in this study, followed by the BAI (EI = 0.652) and the BDI (EI = 0.537) nodes. However, the TAS‐EOT (EI = −0.530), AQ (EI = −0.136), and MAIA (EI = −0.084) showed low values of EI in the network. Regarding the index of strength, the MAIA (strength = 1.273), cognitive empathy (strength = 1.145), the TAS‐DDF (strength = 1.124), and the TAS‐DIF (strength = 1.111) showed the highest values. The detailed values of EI and strength of all the nodes are shown in Table S3.

In the *supplementary* network, the MAIA‐attention‐regulation node (EI = 1.265) had the highest EI, followed by the MAIA‐self‐regulation node (EI = 1.241) and the TAS‐DDF node (EI = 1.235). Regarding the index of strength, the AQ‐communication (strength = 1.133), the MAIA‐self‐regulation (strength = 1.055), and emotion‐awareness (strength = 0.970) showed the highest values. The detailed values of EI and strength in the *supplementary* network can be found in Table S4.

### 3.4 Network Stability

**FIGURE 2:**
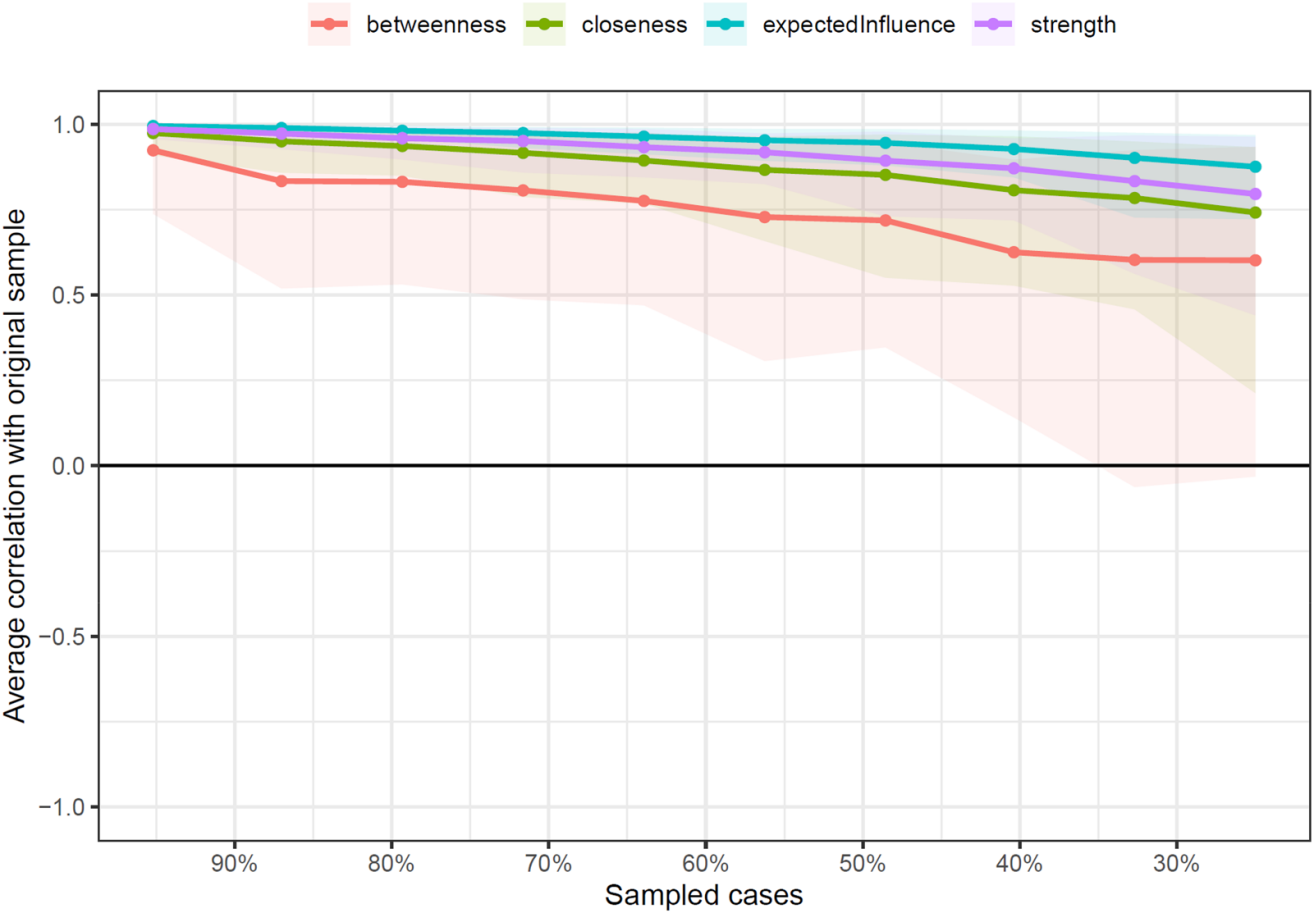
The average correlations between centrality indices of network sampled with persons dropped and the original sample. The *y*‐axis represents the correlation between the centrality index in the original network and that in the network constructed using the bootstrapping method based on a proportion of the original sample. The *x*‐axis represents the percentage of the sampled population in descending order. The dots indicate the correlation coefficients between the values of the centrality index of the two networks. Shadowed areas represent 95% CI of the correlation coefficients.

Figure 2 shows the CS coefficients of the four centrality indices, that is, the maximum dropped proportions to retain correlation of 0.7 in at least 95% of the samples. The results showed that EI had high CS coefficients of 0.75, which lied well above the recommended threshold of 0.5 (Epskamp et al. 2017), supporting excellent stability. The index of strength had an acceptable CS coefficient of 0.60, supporting reasonable stability. However, the indices of closeness and betweenness only had a CS coefficient of 0.44 and 0.05 respectively, that is, falling below the recommended threshold, indicating unstable estimates. As expected, the *supplementary* network had lower network stability as compared to the main results. The CS coefficients of EI, strength, betweenness and closeness had dropped to 0.60, 0.60, 0.05, and < 0.01 respectively. The stability for the indices of EI and strength remained acceptable.

## 4 Discussion

To the best of our knowledge, this is the first network analysis to explore the inter‐relationships among alexithymia, interoception, empathy, self‐awareness, and psychopathological symptoms in young people with ASD. Notably, the TAS‐DDF node demonstrated the highest expected influence, along with a high value of the centrality index of strength, in contrast to the AQ, which had low values in both centrality indices. Such findings suggested that social‐affective difficulties in people with ASD may be influenced by co‐occurring alexithymia more than autistic symptoms, consistent with the alexithymia hypothesis (Bird and Cook 2013). Alexithymia also strongly influenced interoception, empathy, affective symptoms, self‐awareness, and autistic features, replicating and extending Yang et al. (2022)'s results in college students. The association between alexithymia and interoception was also consistent with previous findings gathered in people with ASD (Shah et al. 2016). Moreover, we found a negative association between autistic features and interoception in our network, consistent with the weak association between the two traits found in Yang et al. (2022)'s network. In addition, our network indicated that both alexithymia and interoception were related to cognitive empathy, concurring with Mul et al. (2018)'s findings. Interestingly, the relationship of alexithymia and cognitive empathy was weaker than the relationship between interoception and cognitive empathy, contrary to Mul et al. (2018)'s study which reported that alexithymia mediated the relationship between interoception and cognitive empathy. In Mul et al. (2018)'s study, multiple domains of interoception were captured. However, in our main analysis, we did not parse the MAIA into multiple subscales as nodes in the network.

This work also provided novel findings regarding the important role of anxiety and depressive symptoms in clinical ASD population. In our main analysis, both anxiety and depressive symptoms were positively associated with alexithymia, consistent with previous findings (Milosavljevic et al. 2015; Oakley et al. 2020; Morie et al. 2019) and the emotion dysregulation model of ASD (Cai et al. 2018). In addition, we found associations between interoceptive ability, anxiety and depressive symptoms in clinical ASD population. Higher interoceptive awareness was associated with lower levels of anxiety and depressive symptoms. Our *supplementary* results corroborated that anxiety and depressive symptoms were linked with different dimensions of interoception, including the MAIA‐attention regulation, self‐regulation, not‐worrying, and trusting. Together, our findings generally concurred with recent findings in non‐ASD samples (see Clemente et al. 2024 for meta‐analysis) that several MAIA subscales—such as attention regulation, self‐regulation, not distracting, and trusting—were inversely related to anxiety symptoms. Notably, another previous study on people without ASD also reported that the trusting subscale of MAIA was inversely related to depressive symptoms (Dunne et al. 2021), similar to our *supplementary* findings.

Given the high centrality of alexithymia in the network, particularly the “difficulty‐describing‐feelings” component, targeting alexithymia—alongside depressive and anxiety symptoms—may yield clinical improvements. This aligns with clinical guidelines (e.g., National Institute for Health and Care Excellence 2012), which emphasize the importance of addressing mood and anxiety symptoms in people with ASD. Previous research suggested that alleviating these symptoms could improve core autistic features, educational performance, and employment outcomes (Lidstone et al. 2014; Hedley et al. 2016; Cai and Richdale 2016). Despite having potential clinical implications, alexithymia remains an under‐addressed trait in ASD. Notably, effective interventions developed for people without ASD may offer promise to address alexithymia in the ASD populations. For example, the Emotion Mapping Activity (EMA) can improve alexithymia by directing people to reflect on somatic experiences as cues for identifying and labeling emotions (Edwards et al. 2018). More research should be conducted to determine whether EMA can effectively target alexithymia and associated symptoms in people with ASD. Moreover, intervening the construct of interception may be impactful in people with ASD, given its high values of centrality indices in the network of this study. It is possible to employ the heartbeat discrimination task (Quadt et al. 2021) to train interoceptive accuracy in autistic people. Moreover, the Mindfulness‐integrated Cognitive Behavioral Therapy (MiCBT) can improve interoceptive ability and relates it to emotional experiences in non‐ASD populations (Francis et al. 2022). Future research should verify the applicability of these interventions to people with ASD, for improving their interoception and associated symptoms.

Anxiety and depressive symptoms are common in young people with ASD (Gotham et al. 2015; Hollocks et al. 2019; Kirsch et al. 2020). In our main analysis, the two psychopathological nodes showed high values of expected influence, and modifying these two variables might bring influential impacts to the traits/symptom network. In clinical settings, these symptoms might have atypical presentations, along with other neurosensory and social‐affective problems associated with ASD (Lord et al. 2022). Clinicians' vigilance is essential for early detection and assessments. While cognitive‐behavioral therapy is effective in treating young people with anxiety and depressive disorders (Oud et al. 2019), modifications are likely necessary for delivering treatments effectively to ASD populations. More research is needed to rigorously evaluate the effectiveness of antidepressants in treating depressive symptoms in people with ASD (Howes et al. 2018).

It is noteworthy that the AQ node showed the lowest value of strength, contrary to Yang et al. (2022)'s prior results in the healthy College student sample. It is plausible that, once the autistic symptoms reached clinical severity, its role in the interplay with interoception, alexithymia, empathy and self‐awareness diminished. This phenomenon in people with ASD may be related to the higher prevalence of depressive and anxiety symptoms (Conner et al. 2020), which may undermine the associations of autistic symptoms with interoception, alexithymia, empathy and self‐consciousness, and have not been accounted for in the previous study (Yang et al. 2022). Psychiatric comorbidities of anxiety and depressive disorders in people with ASD may potentially disrupt the relationships between autistics features, alexithymia and interoception. In fact, the mean scores of 21‐item BDI and BAI scores of our sample indicated the presence of mild depressive and anxiety symptoms, and 18% of the sample had comorbid anxiety disorder or depression, consistent with the literature (Kirsch et al. 2020; Lai et al. 2019). Although the AQ communication and several interceptive dimensions (i.e., self‐regulation, attention‐regulation, and emotion‐awareness) have high centrality values in the *supplementary* network, these findings must be interpreted with caution.

This study has several notable limitations. First, our cross‐sectional design precluded the possibility to infer causality. Future research can conduct longitudinal network analysis to clarify the evolution of network structure over time. Second, although the CS‐coefficients of EI and strength indicated stable estimates, our sample size was small and large edge weights in a regularized partial correlation network constructed using a modest sample did not clearly indicate the presence of association between two variables (Huth et al. 2025). Having said that, the present unique sample of ASD, though relatively small, was reasonably sufficient and valuable to conduct a network analysis. Future study should recruit a larger sample to verify the present findings. Third, we only administered self‐report questionnaires to measure the traits and symptoms of interest. Behavioral paradigms should be applied in future research to measure interoception and emotion processing. Fourth, we did not compare the network structure between ASD individuals, subclinical autistic individuals, and neurotypical individuals. Lastly, we did not include social functioning as a node in network analysis. Therefore, it remains unclear how the co‐occurring traits/symptoms we studied could affect social functioning in clinical ASD population. Future research should include this important node, and apply network analysis to identify the putative intervention targets for improving the functional outcomes of ASD.

## 5 Conclusion

This network analysis study demonstrated the close inter‐relationship between alexithymia, interoception, empathy, self‐awareness, depressive and anxiety symptoms in people with ASD. Alexithymia and interoceptive awareness interact substantially with depressive symptoms, anxiety symptoms, and empathy. Intervening ASD individuals' alexithymia and interoception may improve depressive and anxiety symptoms.

## Author Contributions

Simon S.Y. Lui and Raymond C.K. Chan conceived the ideas. Antonio Kei‐Fung Shek and Simon S.Y. Lui designed this study. Antonio Kei‐Fung Shek collected the data; Jason L.F. Chan and Kimi H.Y. Lam assisted with data collection and data management. Antonio Kei‐Fung Shek, Raisie W.K. Wong, and Simon S.Y. Lui conducted the formal analysis. Lok‐Yin Choi assisted in data analysis. Raymond C.K. Chan provided expertise in data interpretation. Antonio Kei‐Fung Shek and Simon S.Y. Lui wrote up the first draft of this manuscript. Eric C.L. Lai, Wang Yi, and Raymond C.K. Chan critically revised the manuscript. Eugenia Y.C. Lok and Siu‐Man Lam provided clinical governance and support. All of the authors commented and approved the final version of the manuscript for submission.

## Funding

Simon S.Y. Lui was supported by HKU Seed Fund for PI Research—Basic Research (Project code: 2302101820). The funding agents have no role in the study design; collection, analysis, and interpretation of the data; writing of the manuscript; nor decision to submit the paper for publication.

## Conflicts of Interest

The authors declare no conflicts of interest.

## Supporting information


**Table S1:** Gender differences in our sample.
**Table S2:** The regularized edges (regularized partial correlation coefficients) in the network.
**Table S3:** The three centrality indices which showed sufficient stability (with CS coefficients > 0.5).
**Table S4:** The two centrality indices which showed sufficient stability (with CS coefficients > 0.5) in the supplementary network.
**Figure S1:** The sample mean and bootstrapped mean of edge weights (with the 95% CIs) in the network.
**Figure S2:** (A) Regularized partial correlation network (with 23 nodes of subscales of measurements) and (B) centrality indices in young adults with ASD (*N* = 208).

## Data Availability

The data that support the findings of this study are available from the corresponding author upon reasonable request.
