# Supplementary material for: A Network Analysis of Alexithymia, Interoception, Empathy, Self‐Awareness and Psychopathological Symptoms in Young People With Autism Spectrum Disorder
Source: Autism Res. 2026 Apr 28;19(6):e70265. doi: 10.1002/aur.70265 (PMC13276700; doi:10.1002/aur.70265)
Supplement: Supplementary file 1 — Table S1: Gender differences in our sample. Table S2: The regularized edges (regularized partial correlation coefficients) in the network. Table S3: The three centrality indices which showed sufficient stability (with CS coefficients > 0.5). Table S4: The two centrality indices which showed sufficient stability (with CS coefficients > 0.5) in the supplementary network. Figure S1: The sample mean and bootstrapped mean of edge weights (with the 95% CIs) in the network. Figure S2: (A) Regularized partial correlation network (with 23 nodes of subscales of measurements) and (B) centrality indices in young adults with ASD (N = 208). [file AUR-19-0-s001.docx]

**Supplementary Materials**

**Supplementary Table 1. Gender differences in our sample**

|  | Male participants  (n = 150) | | Female participants  (n = 58) | | |  |
| --- | --- | --- | --- | --- | --- | --- |
|  | Mean | SD | Mean | SD | t (206) | p |
| Age (years) | 18.56 | 2.907 | 18.60 | 2.871 | -0.097 | 0.923 |
| ADOS-2 SA subscale | 8.13 | 1.149 | 8.09 | 1.048 | 0.233 | 0.816 |
| ADOS-2 RRB subscale | 3.60 | 0.811 | 3.59 | 0.726 | 0.113 | 0.910 |
| AQ-social skill | 5.97 | 2.886 | 6.12 | 2.287 | -0.364 | 0.716 |
| AQ-attention switching | 6.57 | 1.926 | 6.74 | 1.421 | -0.604 | 0.547 |
| AQ-attention to details | 6.00 | 2.192 | 5.69 | 2.226 | 0.912 | 0.363 |
| AQ-communication | 5.67 | 2.527 | 5.76 | 1.994 | -0.249 | 0.804 |
| AQ-imagination | 5.62 | 2.574 | 5.31 | 2.408 | 0.792 | 0.429 |
| TAS-DDF | 16.41 | 4.994 | 16.40 | 4.284 | 0.023 | 0.982 |
| TAS-DIF | 21.72 | 7.532 | 22.41 | 6.852 | -0.610 | 0.542 |
| TAS-EOT | 22.87 | 5.560 | 22.12 | 5.288 | 0.887 | 0.376 |
| SCS score | 36.01 | 11.825 | 37.79 | 10.097 | -1.016 | 0.311 |
| MAIA score | 77.13 | 32.047 | 76.47 | 30.814 | 0.136 | 0.892 |
| QCAE-cognitive | 47.50 | 12.958 | 47.40 | 11.217 | 0.054 | 0.957 |
| QCAE-affective | 26.68 | 6.263 | 29.69 | 6.311 | -3.101 | **0.002** |
| BDI | 15.60 | 10.841 | 17.21 | 11.007 | -0.955 | 0.341 |
| BAI | 15.57 | 11.608 | 17.05 | 11.599 | -0.824 | 0.411 |

Note: BDI = The Beck Depression Inventory ; BAI = The Beck Anxiety Inventory; TAS-DDF = The Toronto Alexithymia Scale- Difficulty Describing Feelings subscale; TAS-DIF = The Toronto Alexithymia Scale- Difficulty Identifying Feelings subscale; TAS-EOT = The Toronto Alexithymia Scale- Externally-Oriented Thinking; MAIA = The Multidimensional Assessment of Interoceptive Awareness; QCAE-cognitive = The Questionnaire for Cognitive and Affective Empathy- cognitive empathy subscale ; QCAE-affective = The Questionnaire for Cognitive and Affective Empathy- affective empathy subscale; SCS = The Self-Consciousness Scale; AQ = Autism-Spectrum Quotient; ADOS-2 = the Autism Diagnostic Observation Schedule Second Edition; SA = the Social Affect subscale of ADOS-2; RRB = the Restricted & Repetitive Behaviors (RRB) subscale of ADOS-2.

**Supplementary Table 2. The regularized edges (regularized partial correlation coefficients) in the network**

|  | BDI | BAI | QCAE-cognitive | QCAE-affective | SCS score | TAS-DDF | TAS-DIF | TAS-EOT | MAIA score | AQ |
| --- | --- | --- | --- | --- | --- | --- | --- | --- | --- | --- |
| BDI | 0 | 0.356 | 0.042 | 0.097 | 0 | 0.187 | 0.023 | 0 | -0.168 | 0 |
| BAI | 0.356 | 0 | 0.065 | 0 | 0.074 | 0.180 | 0.066 | 0 | -0.123 | 0.039 |
| QCAE-cognitive | 0.042 | 0.065 | 0 | 0.065 | 0.132 | 0 | -0.113 | -0.188 | 0.340 | -0.140 |
| QCAE-affective | 0.097 | 0 | 0.065 | 0 | 0.235 | -0.114 | 0 | -0.187 | 0.123 | 0 |
| SCS score | 0 | 0.074 | 0.132 | 0.235 | 0 | 0 | 0 | -0.345 | 0.072 | -0.038 |
| TAS-DDF | 0.187 | 0.175 | 0 | -0.114 | 0 | 0 | 0.489 | 0.124 | -0.021 | 0.014 |
| TAS-DIF | 0.023 | 0.066 | -0.113 | 0 | 0 | 0.489 | 0 | 0.087 | -0.209 | 0.124 |
| TAS-EOT | 0 | 0 | -0.188 | -0.187 | -0.345 | 0.124 | 0.087 | 0 | -0.021 | 0 |
| MAIA score | -0.168 | -0.123 | 0.340 | 0.123 | 0.072 | -0.021 | -0.209 | -0.021 | 0 | -0.136 |
| AQ | 0 | 0.039 | -0.140 | 0 | -0.038 | 0.014 | 0.124 | 0 | -0.136 | 0 |

Note: BDI = The Beck Depression Inventory ; BAI = The Beck Anxiety Inventory; TAS-DDF = The Toronto Alexithymia Scale- Difficulty Describing Feelings subscale; TAS-DIF = The Toronto Alexithymia Scale- Difficulty Identifying Feelings subscale; TAS-EOT = The Toronto Alexithymia Scale- Externally-Oriented Thinking; MAIA = The Multidimensional Assessment of Interoceptive Awareness; QCAE-cog = The Questionnaire for Cognitive and Affective Empathy- cognitive empathy subscale ; QCAE-affective = The Questionnaire for Cognitive and Affective Empathy- affective empathy subscale; SCS = The Self-Consciousness Scale; AQ = Autism-Spectrum Quotient.

**Supplementary Table 3. The three centrality indices which showed sufficient stability (with CS coefficients > 0.5)**

| Node | Expected influence | Strength |
| --- | --- | --- |
| BDI | 0.53656 | 0.87301 |
| BAI | 0.65191 | 0.89858 |
| QCAE-cognitive | 0.26368 | 1.14463 |
| QCAE-affective | 0.21837 | 0.82001 |
| SCS | 0.12979 | 0.89532 |
| TAS-DDF | 0.85445 | 1.12364 |
| TAS-DIF | 0.46666 | 1.11136 |
| TAS-EOT | -0.52941 | 0.95206 |
| MAIA | -0.08422 | 1.27290 |
| AQ | -0.13628 | 0.49051 |

Note: BDI = The Beck Depression Inventory ; BAI = The Beck Anxiety Inventory; TAS-DDF = The Toronto Alexithymia Scale- Difficulty Describing Feelings subscale; TAS-DIF = The Toronto Alexithymia Scale- Difficulty Identifying Feelings subscale; TAS-EOT = The Toronto Alexithymia Scale- Externally-Oriented Thinking; MAIA = The Multidimensional Assessment of Interoceptive Awareness; QCAE-cognitve = The Questionnaire for Cognitive and Affective Empathy- cognitive empathy subscale ; QCAE-affective = The Questionnaire for Cognitive and Affective Empathy- affective empathy subscale; SCS = The Self-Consciousness Scale; AQ = Autism-Spectrum Quotient; EI = the centrality index of expected influence.

**Supplementary Table 4. The two centrality indices which showed sufficient stability (with CS coefficients > 0.5) in the supplementary network**

| Node | Expected Influence | Strength |
| --- | --- | --- |
| ADOS_2_SA | 0.34276 | 0.55420 |
| ADOS_2_RRB | 0.07782 | 0.09820 |
| BDI_total | 0.45555 | 0.72967 |
| BAI_total | 0.44181 | 0.93910 |
| QCAE_cognitive | 0.22398 | 1.14042 |
| QCAE_affective | 0.30573 | 0.83165 |
| AQ_social_skill | 0.31743 | 0.96974 |
| AQ_attention_switching | 0.16053 | 0.6851 |
| AQ_attention_to_details | 0.42694 | 0.69692 |
| AQ_communication | 1.13310 | 1.13310 |
| AQ_imagination | 0.63044 | 0.93214 |
| SCS_total | 0.09828 | 0.98212 |
| TAS_DDF | 0.73499 | 1.12908 |
| TAS_DIF | 0.23644 | 1.23520 |
| TAS_EOT | -0.4485 | 0.99084 |
| MAIA_noticing | 0.15102 | 0.92635 |
| MAIA_not_distracting | -0.0498 | 0.59943 |
| MAIA_not_worrying | -0.0484 | 0.62365 |
| MAIA_attention_regulation | 0.45296 | 1.26502 |
| MAIA_emotion_awareness | 0.97026 | 1.07314 |
| MAIA_self_regulation | 1.05479 | 1.24104 |
| MAIA_body_listening | 0.86035 | 0.99996 |
| MAIA_trusting | 0.32329 | 1.03052 |

Note: ADOS_2_SA = The Autism Diagnostic Observation Schedule Second Edition Module 4-Social Affect subscale; ADOS_2_RRB = The Autism Diagnostic Observation Schedule Second Edition Module 4-the Restricted & Repetitive Behaviors subscale; BDI_total = The Beck Depression Inventory; BAI_total = The Beck Anxiety Inventory; QCAE_cognitive = The Questionnaire for Cognitive and Affective Empathy-cognitive empathy subscale; QCAE_affective = The Questionnaire for Cognitive and Affective Empathy-affective empathy subscale; AQ_social_skill = The Autism-Spectrum Quotient-social skills scale; AQ_attention_switching = The Autism-Spectrum Quotient-attention switching scale; AQ_attention_to_details = The Autism-Spectrum Quotient-attention-to-detail scale; AQ_communication = The Autism-Spectrum Quotient-communication scale; AQ_imagination = The Autism-Spectrum Quotient-imagination scale; SCS_total = The Self-Consciousness Scale; TAS_DDF = The Toronto Alexithymia Scale-Difficulty Describing Feelings subscale; TAS_DIF = The Toronto Alexithymia Scale-Difficulty Identifying Feelings subscale; TAS_EOT = The Toronto Alexithymia Scale-Externally-Oriented Thinking; MAIA_noticing = The Multidimensional Assessment of Interoceptive Awareness-Noticing subscale; MAIA_not_distracting = The Multidimensional Assessment of Interoceptive Awareness-Not-distracting subscale; MAIA_not_worrying = The Multidimensional Assessment of Interoceptive Awareness-Not-worrying subscale; MAIA_attention_regulation = The Multidimensional Assessment of Interoceptive Awareness-Attention regulation subscale; MAIA_emotion_awareness = The Multidimensional Assessment of Interoceptive Awareness-Emotional awareness subscale. MAIA_self_regulation = The Multidimensional Assessment of Interoceptive Awareness-Self-regulation subscale; MAIA_body_listening = The Multidimensional Assessment of Interoceptive Awareness-Body listening subscale; MAIA_trusting = The Multidimensional Assessment of Interoceptive Awareness-Trusting subscale.

**Supplementary Figure 1. The sample mean and bootstrapped mean of edge weights (with the 95% Cis) in the network**

**
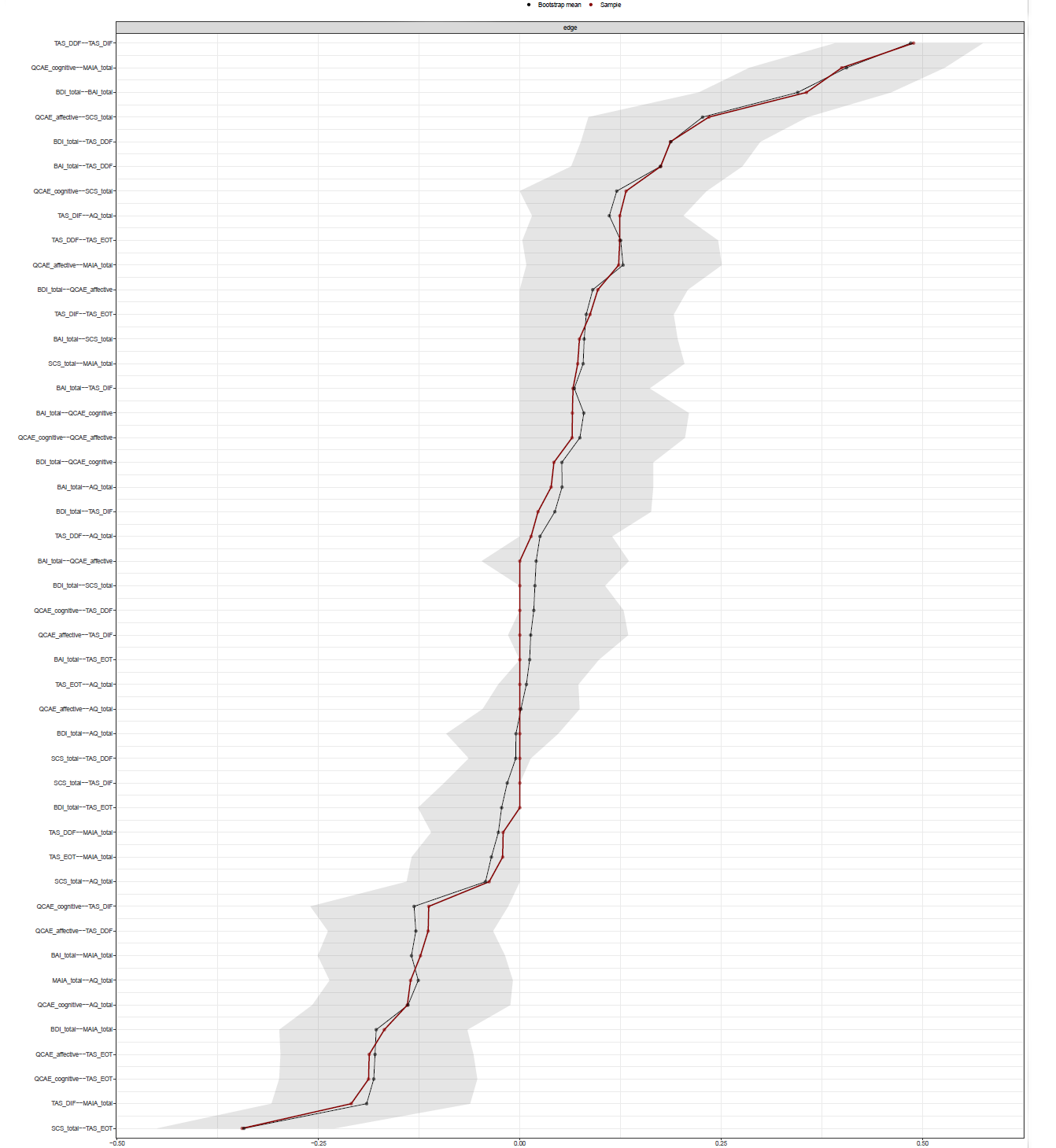
**

The black dots indicate the sample mean, whereas the red black dots indicate the bootstrapping mean, for each of the edge weight. The shadowed area shows the range of R falling within the 95% CI. Edge having a R with 95% CI above and below zero indicates better stability.

**Supplementary Figure 2. (A) Regularized partial correlation network (with 23 nodes of subscales of measurements) and (B) centrality indices in young adults with ASD (N = 208)**

**A**


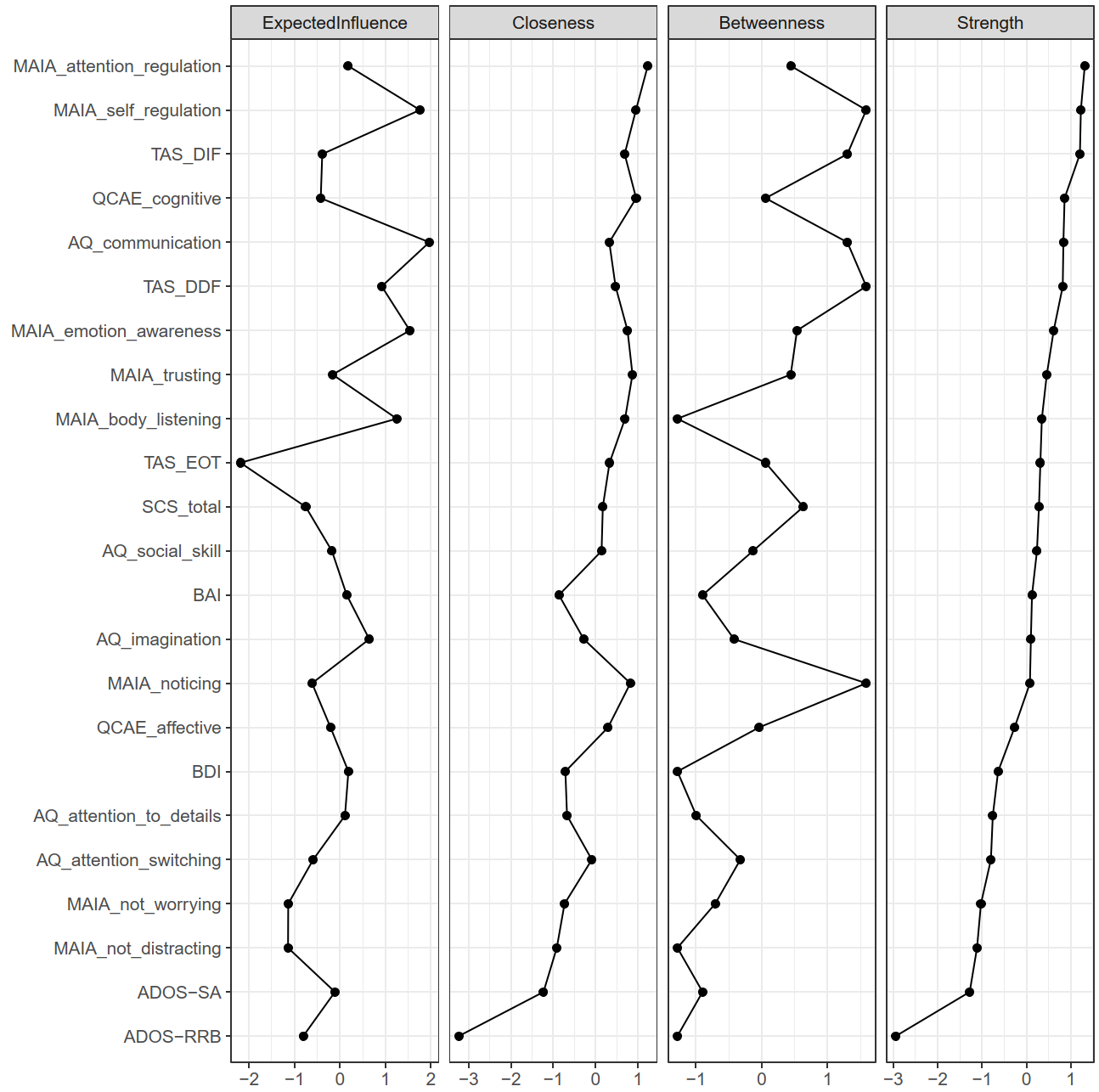


**B**

Notes: The green lines are positive associations. Red lines are negative associations. Thickness and saturation of lines are the strength of associations. The filled part of the circle around each node is the predictability. ADOS_2_SA = The Autism Diagnostic Observation Schedule Second Edition Module 4-Social Affect subscale; ADOS_2_RRB = The Autism Diagnostic Observation Schedule Second Edition Module 4-the Restricted & Repetitive Behaviors subscale; BDI_total = The Beck Depression Inventory; BAI_total = The Beck Anxiety Inventory; QCAE_cognitive = The Questionnaire for Cognitive and Affective Empathy-cognitive empathy subscale; QCAE_affective = The Questionnaire for Cognitive and Affective Empathy-affective empathy subscale; AQ_social_skill = The Autism-Spectrum Quotient-social skills scale; AQ_attention_switching = The Autism-Spectrum Quotient-attention switching scale; AQ_attention_to_details = The Autism-Spectrum Quotient-attention-to-detail scale; AQ_communication = The Autism-Spectrum Quotient-communication scale; AQ_imagination = The Autism-Spectrum Quotient-imagination scale; SCS_total = The Self-Consciousness Scale; TAS_DDF = The Toronto Alexithymia Scale-Difficulty Describing Feelings subscale; TAS_DIF = The Toronto Alexithymia Scale-Difficulty Identifying Feelings subscale; TAS_EOT = The Toronto Alexithymia Scale-Externally-Oriented Thinking; MAIA_noticing = The Multidimensional Assessment of Interoceptive Awareness-Noticing subscale; MAIA_not_distracting = The Multidimensional Assessment of Interoceptive Awareness-Not-distracting subscale; MAIA_not_worrying = The Multidimensional Assessment of Interoceptive Awareness-Not-worrying subscale; MAIA_attention_regulation = The Multidimensional Assessment of Interoceptive Awareness-Attention regulation subscale; MAIA_emotion_awareness = The Multidimensional Assessment of Interoceptive Awareness-Emotional awareness subscale; MAIA_self_regulation = The Multidimensional Assessment of Interoceptive Awareness-Self-regulation subscale; MAIA_body_listening = The Multidimensional Assessment of Interoceptive Awareness-Body listening subscale; MAIA_trusting = The Multidimensional Assessment of Interoceptive Awareness-Trusting subscale.

**Syntax of the main analysis**

### Load packages ###

library(Hmisc)

library(bootnet)

library(qgraph)

library(networktools)

library(NetworkComparisonTest)

library(mgm)

library(bnlearn)

library(e1071)

library(Rgraphviz)

library(igraph)

library(huge)

rm(list=ls())

library(readxl)

library(bootnet)

library(qgraph)

library(huge)

### Load 10 nodes data into R ###

data <-read.table("Antonio_data_20251217.csv",header=TRUE,sep=",",na=" ",as.is=T)

head(data)

colnames(data)

### Aesthetics for graphs ###

data<-subset(data,select = BDI:MAIA) # nodes = 10

data$AQ <-rowSums(data[,c("AQ_social_skill","AQ_attention_switching","AQ_attention_to_details","AQ_communication","AQ_imagination")]

data <- data[,c((1:4),(10:15))]

## Aesthetics for graphs ##

longnames <- c(

"BDI_total",

"BAI_total",

"QCAE_cognitive",

"QCAE_affective",

"SCS_total",

"TAS_DDF",

"TAS_DIF",

"TAS_EOT",

"MAIA_total",

"AQ_total")

clusters<-list("Depression"=c(1),"Anxiety"=c(2), "QCAE"=c(3:4),"SCS"=c(5),"TAS"=c(6:8), "MAIA"=c(9),"AQ"=c(10))

### Estimate regularised partial correlation networks ###

NET <- estimateNetwork(data,default="EBICglasso",corMethod="npn")

NET$graph # Correlation Matrix

### Get the Correlation Matrix ###

write.csv(NET$graph,file = "anthonio_corrmatrix.csv")

## Compute centrality indices ###

cent <- centrality(NET)

### Print centrality indices into PDF ###

pdf("Centrality.pdf", width=8, height=8)

centralityPlot <- centralityPlot(NET, scale="z-scores", include = c("ExpectedInfluence","Closeness","Betweenness","Strength"),

labels = longnames, orderBy = "Strength")

centralityPlot + theme(

axis.text = element_text(size = 14)

)

dev.off()

### Edge weight bootstrap ###

set.seed(888)

boot_edge <- bootnet(NET, default="EBICglasso", nCores = 8, nBoots = 2500,

statistics = c("edge","expectedInfluence","strength"),

type = "nonparametric")

### Save edge weight bootstrap ###

save(boot_edge, file = "boot_edge.Rdata")

### Print edge weight bootstrap into PDF ###

pdf("boot_edge.pdf", width=21, height=24)

plot(boot_edge, labels = TRUE, order = "sample")

dev.off()

### Centrality stability ###

### Case-dropping bootstrap ###

set.seed(888)

boot_case <- bootnet(NET, default ="EBICglasso", nCores = 8, nBoots= 1000,

statistics = c("strength","expectedInfluence","closeness","betweenness","edge"),

type = "case")

### Compute CS-coefficients ###

corStability(boot_case)

### Save case-droppping bootstrap ###

save(boot_case, file = "boot_case.Rdata")

write.csv(corStability(boot_case),file = "corStability.csv")

### Print boot_case into pdf ###

pdf("boot_case.pdf", width=7, height=5)

plot(boot_case, statistics = c("strength","expectedInfluence","closeness","betweenness"), labels = TRUE, order = "sample")

dev.off()

### Testing for significant differences ###

### Print significant differences of edge weights into pdf ###

pdf("Diff_EDGE.pdf", width = 20, height =20)

plot(boot_edge, "edge", plot = "difference", onlyNonZero = TRUE, cez.axis =0.5, order = "sample")

dev.off()

### Print significant differences of node expected influence into pdf ###

pdf("Diff_EXPECTEDINFLUENCE.pdf", width=6, height=6)

plot(boot_edge, "expectedInfluence")

dev.off()

### Print significant differences of node strength into pdf ###

pdf ("Diff_STRENGTH.pdf", width=6, height=6)

plot(boot_edge, "strength")

dev.off()

### Convert qgraph object to igraph object ###

q <- qgraph(NET$graph, labels=colnames(data), layout="spring",

vsize=6, cut=0, border.width=1.5, border.color="black",

posCol="blue",

groups=clusters,

nodeNames = longnames)

### Re-estimate networks via mgm and compute the node predictability ###

data_matrix <- as.matrix(data)

type = rep('g', ncol(data_matrix))

level <- rep(1, ncol(data_matrix))

fit <- mgm(na.omit(data_matrix), type = type, level = level, k=2)

pred <- predict(fit, na.omit(data_matrix), error.continuous = 'VarExpl')

pred$errors

mean(pred$errors[,3])

### Get the Centrality Index ###

write.csv(cent$InDegree,file = "Strength.csv")

write.csv(cent$InExpectedInfluence,file = "ExpectedInfluence.csv")

write.csv(cent$Closeness,file = "Closeness.csv")

write.csv(cent$Betweenness,file = "Betweenness.csv")

write.csv(pred$errors,file = "Predictability.csv")

### Print the networks into PDF ###

pdf("NET_C.pdf", width=22, height=15)

qgraph(NET$graph,

labels = colnames(data),

layout = 'circle',

palette = 'colorblind', # Choose a colorblind-friendly colour palette

theme = 'colorblind', # Choose a colorblind-friendly colour palette

vsize = 9, # Makes the circle larger

cut = 0,

border.width = 1.5,

border.color = "black",

posCol = "green",

groups = clusters,

nodeNames = longnames,

pie = pred$errors[,3],

label.cex = 1.2, # Makes the text font larger

label.prop = 0.6, # Allows text to fill 60% of the node area

label.font = 1, # Makes the labels not bold

legend.cex = 1.0 # Make the legends larger

)

dev.off()

pdf("NET_S.pdf", width=22, height=15)

qgraph(NET$graph,

labels = colnames(data),

layout = 'spring',

palette = 'colorblind', # Choose a colorblind-friendly colour palette

theme = 'colorblind', # Choose a colorblind-friendly colour palette

vsize = 9, # Makes the circle larger

cut = 0,

border.width = 1.5,

border.color = "black",

posCol = "green",

groups = clusters,

nodeNames = longnames,

pie = pred$errors[,3],

label.cex = 1.2, # Makes the text font larger

label.prop = 0.6, # Allows text to fill 60% of the node area

label.font = 1, # Makes the labels not bold

legend.cex = 1.0 # Make the legends larger

)

dev.off()
